# Immunohistochemical Evaluation of Peri-Implant Soft Tissues around Machined and Direct Metal Laser Sintered (DMLS) Healing Abutments in Humans

**DOI:** 10.3390/ijerph15081611

**Published:** 2018-07-30

**Authors:** Carlo Mangano, Francesco Guido Mangano, Jamil Awad Shibli, Leandro Amadeu Roth, Gianmaria d’ Addazio, Adriano Piattelli, Giovanna Iezzi

**Affiliations:** 1 Department of Dental Sciences, Dental School, San Raffaele University, Milan 20132, Italy; 2 Department of Medicine and Surgery, Dental School, Insubria University, Varese 21100, Italy; francescoguidomangano@gmail.com; 3 Department of Periodontology and Oral Implantology, Dental Research Division, Guarulhos University, Guarulhos 743372, SP, Brazil; jashibli@yahoo.com (J.A.S.); rothleandro@gmail.com (L.A.R.); 4 Department of Medical, Oral and Biotechnological Sciences, University G. d’Annunzio, Chieti 66100, Italy; gianmariad@gmail.com (G.d’.A.); apiattelli@unich.it (A.P.); gio.iezzi@unich.it (G.I.); 5 Catholic University of San Antonio de Murcia (UCAM), Murcia 30107, Spain

**Keywords:** healing abutment, soft tissues, machined, direct metal laser sintering, humans

## Abstract

***Background***: Direct metal laser Sintering (DMLS) is an additive manufacturing technique that allows fabrication of dental implants and related components with a highly porous surface. To date, no human studies have investigated the soft tissue adhesion and presence of inflammatory infiltrate with porous DMLS healing abutments (HAs), nor have they compared these with the classic machined ones. ***Purpose***: To evaluate the degree of cell adhesion (integrin expression) and the quantity/quality of inflammatory infiltrate, on HAs with different surfaces; full DMLS, full machined, and hybrid (half DMLS and half machined). ***Methods***: Fifty implant patients were randomly assigned to receive one of these different Has: T1, full DMLS (11 subjects); T2, machined in the upper portion and DMLS in the lower one (10 subjects); T3, DMLS in the upper portion and machined in the lower one (19 subjects); T4, full machined (10 patients). Thirty days after placement, circular sections of soft tissues around HAs were retrieved for immunohistochemical evaluation. ***Results***: With regard to the adhesion molecules, the samples showed different intensity of integrin expression, with a statistically significant difference (*p* < 0.001) between T1 and the other groups. All the samples were positive for the different clusters related to the inflammatory infiltrate (T lymphocytes, CD3; B lymphocytes, CD20; and macrophages, CD68), but a lower infiltrate was found in T1, with statistically significant differences (*p* < 0.001) among the groups. ***Conclusions:*** The HA surface seems to influence the degree of cell adhesion and the inflammatory infiltrate of the surrounding soft tissues.

## 1. Introduction

Material and surface treatment of the transmucosal portion of implant rehabilitation can play a role in the long-term maintenance of dental implants [1]. In fact, they can counteract or delay plaque accumulation, and the quantity/quality of adherent bacteria [1,2]. It is well known that bacteria accumulation and stable biofilm formation induce the development of inflammatory lesions and soft/hard tissues inflammation [3,4,5,6]. At the same time, it is important to create a soft tissue barrier around the implant, able to protect the underlying bone structure [1,4,7,8,9,10,11,12]. It is well known that the soft tissues play a relevant role in the pathogenesis and progression of peri-implant pathology [4,5,6,7,8,9,10,11,12].

The literature has demonstrated that the surface topography of the abutment can influence the formation of biological width [12,13,14]. A rough surface can positively influence the attachment of cells [15,16,17]. The cell binding on the surface is mediated by the formation of hemidesmosomes, as in tooth [12,13,15,16,17,18]. The surface treatment could, therefore, influence the quality/quantity of these attachments as well as the healing process and host response [1,15,16,17].

Over the years, several techniques have been used to modify and treat the implant surface and studies about different treatments of the transgingival portion have been conducted [18,19,20,21]. From these studies, it seems that surfaces with homogeneous roughness can favor the epithelial and connective attachment and, therefore, the formation of a soft tissue barrier [1,18,19,20,21].

Recently, a new additive manufacturing technique, namely direct metal laser sintering (DMLS), has been introduced, offering a new venue for treatment of implant and abutment surfaces [22,23,24] Among the several metal forming techniques, DMLS offers potential benefits in the field of implant dentistry, because of its capability to directly build three-dimensional (3D) components from metal (titanium) powders with minimal or no post-processing requirements [22,23,24]. By means of a high energy focused laser beam, a localized region of a thin layer of metal powder directly melts, in accordance with a sliced 3D computer aided design (CAD) model [22,23,24]. Using this additive manufacturing technique, it is possible to fabricate 3D freeform objects, including dental implants, directly from CAD models [23]. The precision of this technique, by which it is possible to melt very thin sections (from 0.02 to 0.06 mm) together, permits very complex titanium geometries to be fabricated, with a highly porous surface and a gradient of porosity along the main axis of the implant [23,24]. The difference of material density introduced thus brings to dentistry a new functionally graded material [23,24].

Several reviews [24], research [25,26,27,28], and clinical studies [29,30,31] have demonstrated the potential advantages and applications of the DMLS technique for the fabrication of dental implants, even with custom shapes [32,33].

Recently, one histological and scanning electron microscope human study has evaluated the organization of the peri-implant soft tissues around DMLS titanium implants, showing an intimate contact of the fibrous matrix with the implant surface, and some collagen fibers that were perpendicularly directed to the laser sintered surface [34].

However, to date, there are no human studies that have investigated the soft tissue adhesion and presence of inflammatory infiltrate with highly porous DMLS healing abutments (Has), and that have compared these with the classic machined ones. Hence, the aim of the present study was to evaluate the degree of cell adhesion (by means of integrins) and the quantity/ quality of inflammatory infiltrate, on HAs with different surfaces; full DMLS porous surface, full machined surface, and hybrid abutments (half DMLS and half machined).

## 2. Materials and Methods

### 2.1. Study Design

A total of 50 biopsy samples taken from the peri-implant soft tissues were collected in order to perform the present immunohistochemical study. The samples came from patients undergoing submerged implant surgery, and the relative prosthetic rehabilitation. Immunohistochemical analysis of soft tissues surrounding HAs were performed. All samples come from the University of Guarulhos, São Paulo, Brazil. All patients received information and instructions regarding the study protocol and each patient signed a written informed consent form. The protocol was approved on 11 November 2008 by the Ethics Committee of the University of Guarulhos (UnG) (protocol number 113/2008). It was registered on Sistema Nacional de Informação Sobre Ética em Pesquisa Envolvendo Seres Humanos (SISNEP) number 379. It was carried out in accordance with the principles set out in the Helsinki Declaration developed by the World Medical Association on Human Experimentation, as revised in 2000.

The inclusion criteria were as follows: age over 18, good systemic and oral health, loss of one to three elements in the posterior regions (premolars and molars) of maxillae or mandibules, keratinized mucosa (KT) width of at least 3 mm, and also a period of at least six weeks of healing after the extraction of the tooth.

The exclusion criteria were as follows: poor oral hygiene, active periodontal disease or other oral disorders, insufficient amount of bone for implant rehabilitation, bone augmentation procedures with autologous bone or bone substitutes, uncontrolled diabetes mellitus, immune diseases, smoking, and bruxism.

**Figure 1:**
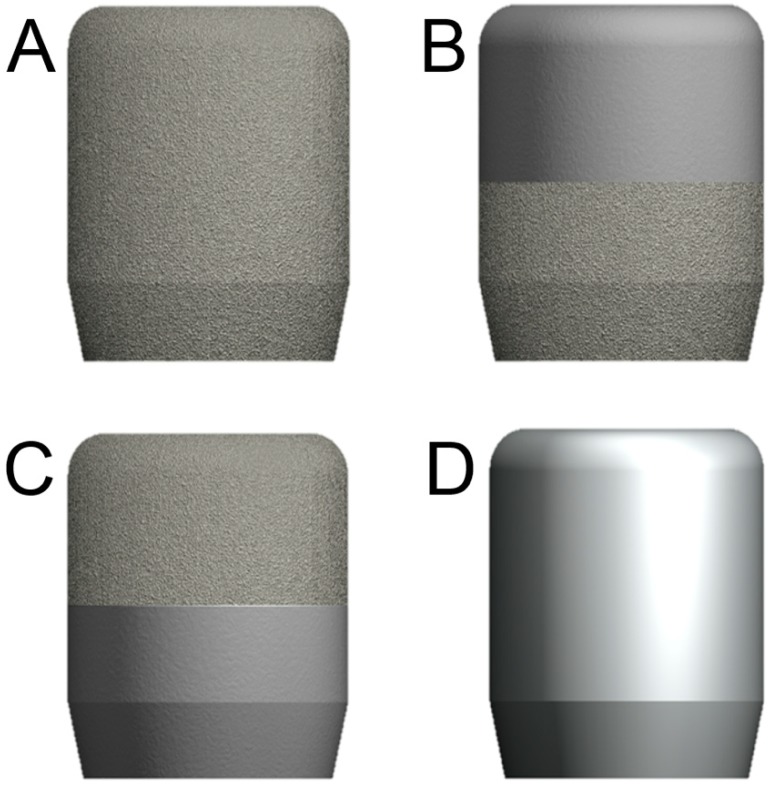
Schematic drawings of the different healing abutments used in this study. (**A**) Direct metal laser sintering (DMLS) healing abutment (T1); (**B**) healing abutment with a smooth surface in the upper half and a DMLS surface in the lower half (T2); (**C**) healing abutment with a DMLS surface in the upper half and a smooth surface in the lower half (T3); (**D**) healing abutment with an entirely smooth (machined) surface.

In brief, after inclusion in the study, each patient was assigned, randomly, to one of four possible groups (T1, T2, T3, and T4) based on the type of HA used. In the T1 group (11 patients), all subjects received a HA with a full DMLS surface (TixOs^®^, Leader Implants, Milan, Italy) (Figure 1A). In the T2 group (10 patients), all subjects received a HA with a smooth surface in its upper half, and a DMLS surface in the lower half portion (Figure 1B). In the T3 group (19 patients), the HA had a DMLS surface in its upper half, and a smooth surface in the lower half portion (Figure 1C). Finally, in the T4 group (10 patients), all subjects received a completely smooth (machined) HA (Figure 1D).

### 2.2. Direct Metal Laser Sintering Implants and Components

All fixtures used in the present study (TixOs^®^, Leader, Milan, Italy) were fabricated as previously described [26,27,28,29,30,31]. In brief, titanium alloy (Ti_6_Al_4_V) powders with a particle size of 25–45 μm were used as basic material in an argon atmosphere, and sintered together, layer by layer, through a DMLS process. The laser machine (Eosynt^®^270, EOS, Munich, Germany) used an ytterbium laser system with a spot size of 0.1 mm, a wavelength of 1054 nm, a continuous power of 200 W at a scanning rate of 7 m/s. The machine had a build-up area of 250 × 250 × 215 mm. After the DMLS process, the fixtures presented a porous surface covered by spherical non-adherent titanium particles. In order to remove these particles, the fixtures were sonicated for 5 min in distilled water at 25 °C, immersed in NaOH (20 g/L) and hydrogen peroxide (20 g/L) at 80 °C for 30 min, and then further sonicated for 5 min in distilled water. Following these processes, an organic acid treatment was performed to further clean the surface, in a mixture of 50% oxalic acid and 50% maleic acid at 80 °C for 45 min, followed by washing for 5 min in distilled water in a sonic bath. As a result of this treatment, all non-adherent residual particles were removed from the implant surface that presented a highly porous aspect, with a mean Ra value of 66.8 μm, a mean Rq value of 77.55 μm, and a mean Rz value of 358.3 μm. The implants used in this study were available in different diameters (3.3 mm, 3.75 mm, 4.1 mm, 4.75 mm) and lengths (10.0 mm, 11.5 mm, 13.0 mm).

The same DMLS procedure was used to produce the HAs T1, which had a complete laser sintered surface. The HAs T2 and T3 had a machined portion without any porosity, in the upper half and in the lower half, respectively, because the laser parameters there were set to produce a dense surface, which was subsequently turned/machined; however, the T2 and T3 HAs had 50% of their surface (in the lower half and in the upper half, respectively) that was laser sintered and porous. Finally, the T4 HAs were fully machined.

### 2.3. Surgical Protocol

For each patient, a complete examination of the hard and soft tissues of the oral cavity was performed and a panoramic radiograph was taken as a first level survey. The implant placement was performed by an expert surgeon in local anesthesia with 4% articaine solution containing 1:100,000 adrenaline, and crestal incision by performing a full-thickness flap and relative dissection. Once the underlying bone profile was exposed, the implant site was prepared with spiral drills of increasing diameter under abundant physiological irrigation, in order to prevent overheating of the bone. At the end of the surgical procedure, the implants were placed, showing a good primary stability; cover caps were screwed on and the fixtures were submerged. Implants were inserted with an average insertion torque of 45 N/cm, measured by a hand-operated key to measure the digital torque. All patients were then subjected to oral antibiotic therapy with 2 gr per day for six days (Augmentin^®^, GLaxo-Smithkline Beecham, Brentford, UK), while post-operative pain was controlled with 100 mg of nimesulide administration (Aulin^®^, Roche Pharmaceutical, Basel, Switzerland) every 12 h for two days. Detailed oral hygiene instructions as well as a 0.20% chlorhexidine rinse (Chlorexidine^®^, Oral-B, Boston, MA, USA) were also provided for seven days. Suture were removed after 8–10 days.

After a healing period of two months for the mandible and four months for the maxilla, during the second surgical phase, the implants were uncovered, the cover caps/screws were removed and replaced with the four different types of HAs (T1 with DMLS surface; T2 with smooth surface in the upper half and a DMLS surface in the lower half; T3 with DMLS surface in the upper half and smooth surface in the lower half; T4 entirely machined). Sutures were performed around the healing abutments. The HAs remained in situ for a period of 30 days.

### 2.4. Primary Outcomes 

The primary outcomes of this immunoistochemical human study were the adhesion of the soft tissue on the HAs, measured by means of integrins α6 expression, and the quality/quantity of inflammatory infiltrate in the specimens, by means of the presence of T lymphocytes (CD3), B lymphocytes (CD20), and macrophages (CD68). Finally, immunohistochemical analysis of the blood vessels was also performed (CD31), investigating their microdensity.

### 2.5. Specimen Retrieval

After this healing period, before starting with the prosthetic restoration of the fixtures, the different HAs and circular sections of soft tissues around them were retrieved for analysis. The size of the gingival biopsies averaged 2.1 mm (5.5–3.8 mm) in thickness and 3 mm in height. All circular punches were made based on the amount of KT and the diameter of the healing abutments. Specifically, the diameter was 1.5 mm wider than the healing screw. A total of 50 samples were retrieved. These specimens were treated for immunohistochemistry. Three-micron sections were obtained with a Leitz 1512 microtome and stained with Hematoxylin–Eosin (H&E). The immunohistochemical staining of integrins α6 (rabbit polyclonal antibody, for human use, in paraffine sections) and CD20, CD3, CD68, and CD31 (specific markers of inflammation) was performed using the strep-ABC (Streptavidine–Biotine–Peroxidase) method (OriGene Technologies, Inc., Rockville, MD, USA). α6 was chosen to evaluate the amount of expression of the integrins as it represents the main component of hemidesmosomes. In this way, it would have been possible to know the expression of the molecule as an index of soft tissue bindings on HAs [35]. Three-micron sections were cut and mounted on poly-l-lysine-coated slides. Paraffin sections were dewaxed by xylene, rehydrated, and finally washed in PBS (pH 7.4) for 10 min. In order to unmask the antigens, a microwave oven and a 2.1% content of citric acid were used related to the antibodies. The subsequent steps were optimized by automatic staining (Optimax^®^, BioGenex, San Ramon, CA, USA)*.* Sections were incubated with primary antibody for 30 min at room temperature. Slides were rinsed in buffer, and immunoreaction was completed with the Strep-ABC-Peroxidase method, applying the “super sensitive immunodetection” kit by BioGenex (San Ramon, CA, USA) and utilizing a multi-link as a secondary biotinylated antibody. After incubation with a chromogen employing “liquid DAB substrate pack” (BioGenex, San Ramon, CA, USA), the specimens were counterstained with Mayer’s hematoxyline and coverslipped. In each sample, five random fields at high magnification (20×) were chosen. The high magnification images were acquired using a dedicated software (QWIN^®^, Leica, Wetzlar, Germany) connected to a light microscope (DMR^®^, Leica, Wetzlar, Germany). The same software (QWIN^®^, Leica, Wetzlar, Germany) allowed us to identify a color gradation in the samples. This colorimetric scale was thus decisive in assigning a percentage of area of the sample under examination (automatically excluding the white areas of the slide). In this way, it was possible to calculate within the area of interest the percentage of positive area at the immunohistochemical stain.

### 2.6. Statistical Analysis

The data obtained were evaluated by the Kruskal–Wellis test; the differences between the groups were calculated using the Dunn multiple comparison test. All data are presented with mean ± standard deviation (SD); a value of *p* ≤ 0.001 was considered statistically significant.

## 3. Results

**Figure 2:**
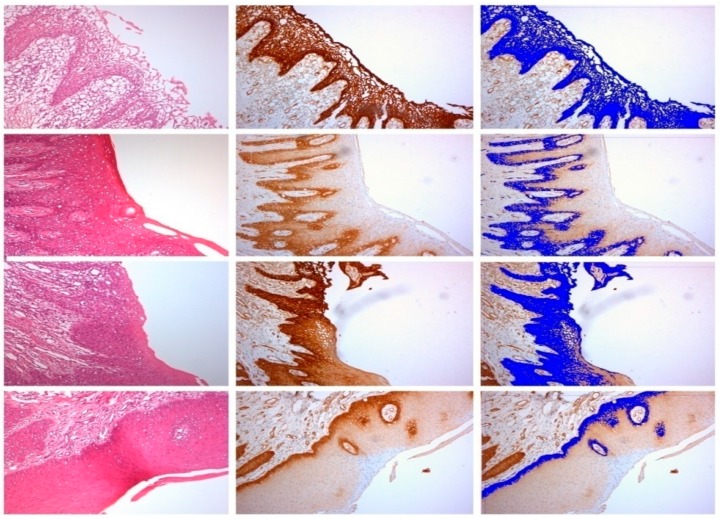
Immunohistochemical expression of integrins. From the top to bottom: T1, T2, T3, T4. The left column shows H&H stain; the second one represents the Integrin alpha6 expression (strep-ABC—Streptavidine–Biotine–Peroxidase); the right column shows software analysis.

**Figure 3:**
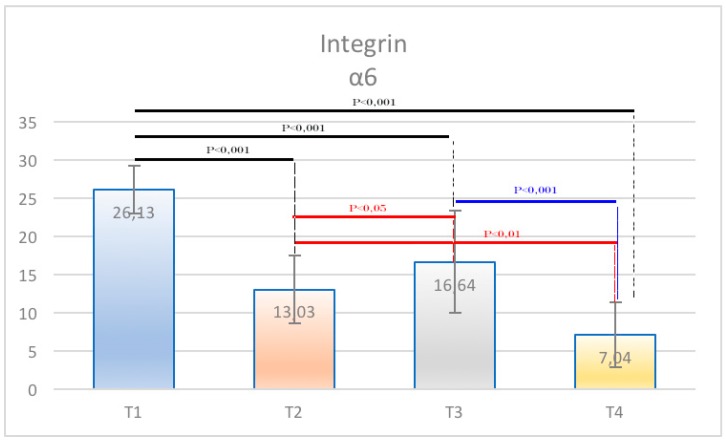
Graphical representation of α6 integrins in different groups. Statistically significant differences between groups were found.

**Figure 4:**
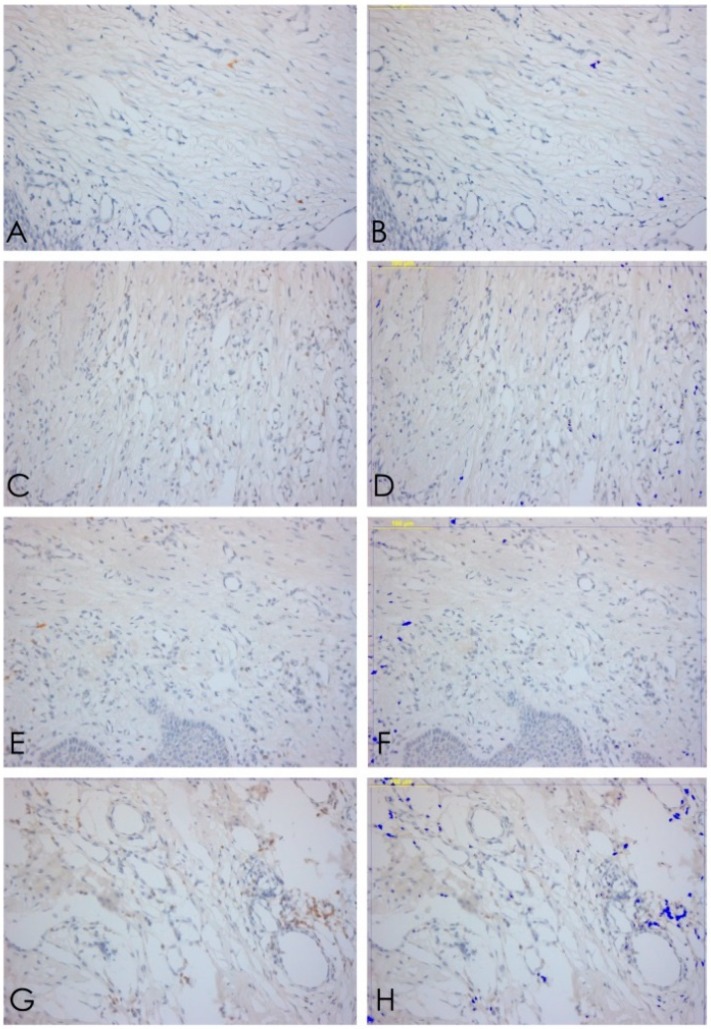
CD3. (**A**,**B**) T lymphocytes expression T1, 20× ABC, and software analysis; (**C**,**D**) T lymphocytes expression T2, 20× ABC, and software analysis; (**E**,**F**) T lymphocytes expression T3, 20× ABC, and software analysis; (**G**,**H**) T lymphocytes expression T4, 20× ABC; and software analysis.

**Figure 5:**
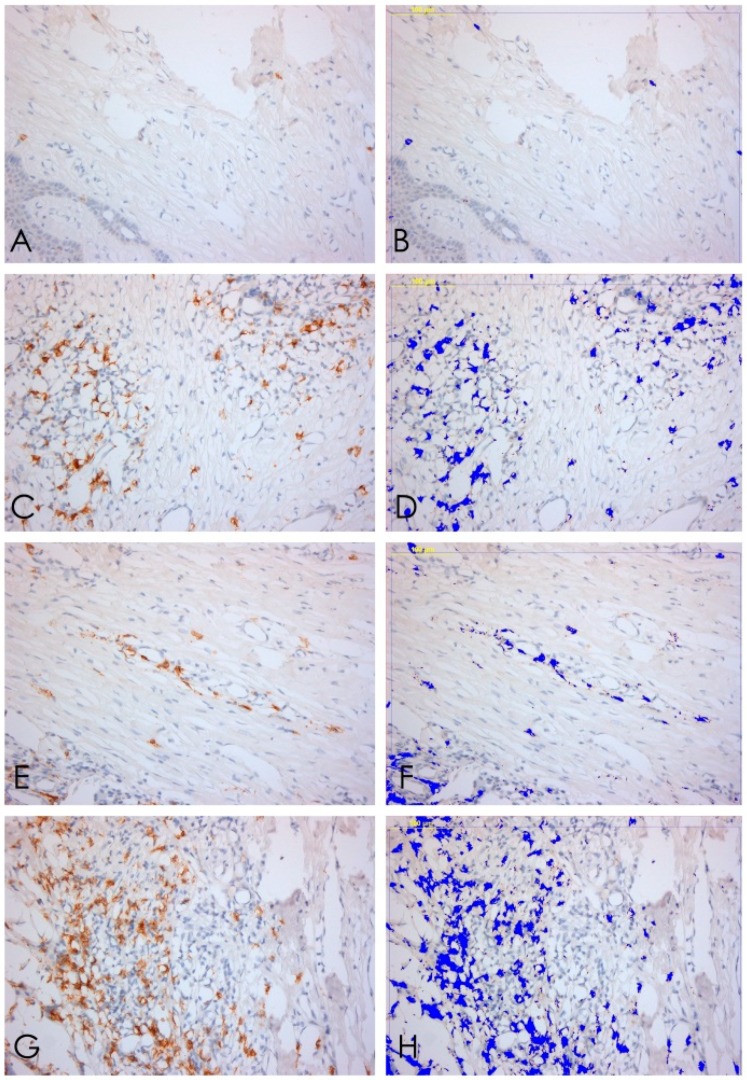
CD20. (**A**,**B**) B lymphocytes expression T1, 20× ABC, and software analysis; (**C**,**D**) B lymphocytes expression T2, 20× ABC, and software analysis; (**E**,**F**) B lymphocytes expression T3, 20× ABC, and software analysis; (**G**,**H**) B lymphocytes expression T4, 20× ABC; and software analysis.

**Figure 6:**
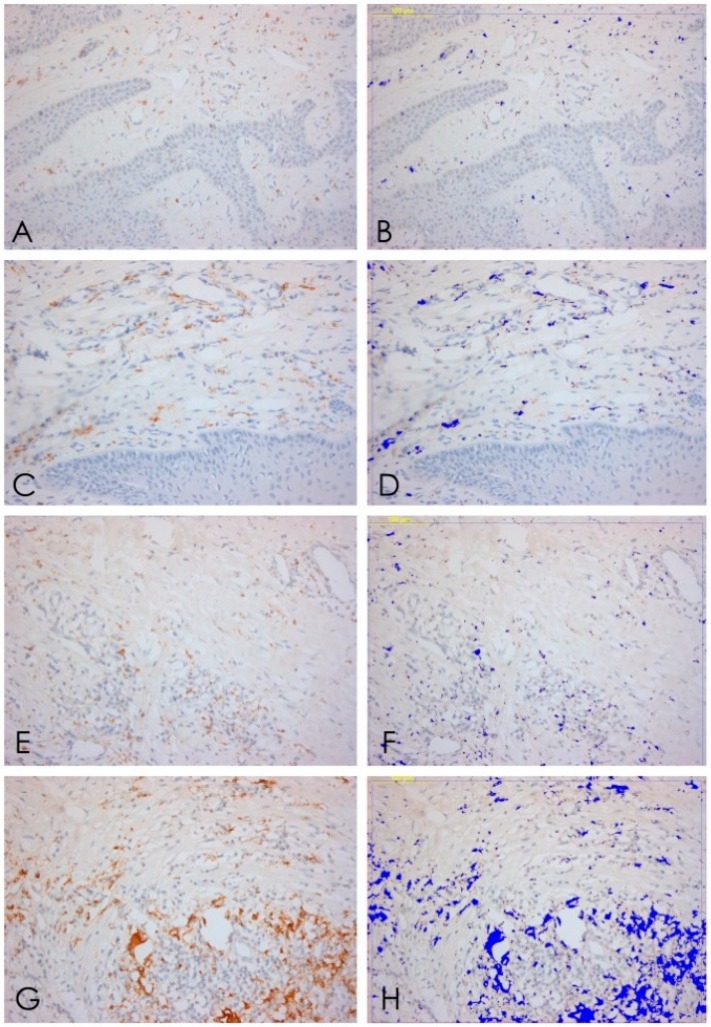
CD68. (**A**,**B**) Macrophages expression T1, 20× ABC, and software analysis; (**C**,**D**) macrophages expression T2, 20× ABC, and software analysis; (**E**,**F**) macrophages expression T3, 20× ABC, and software analysis; (**G**,**H**) macrophages expression T4, 20× ABC, and software analysis.

**Figure 7:**
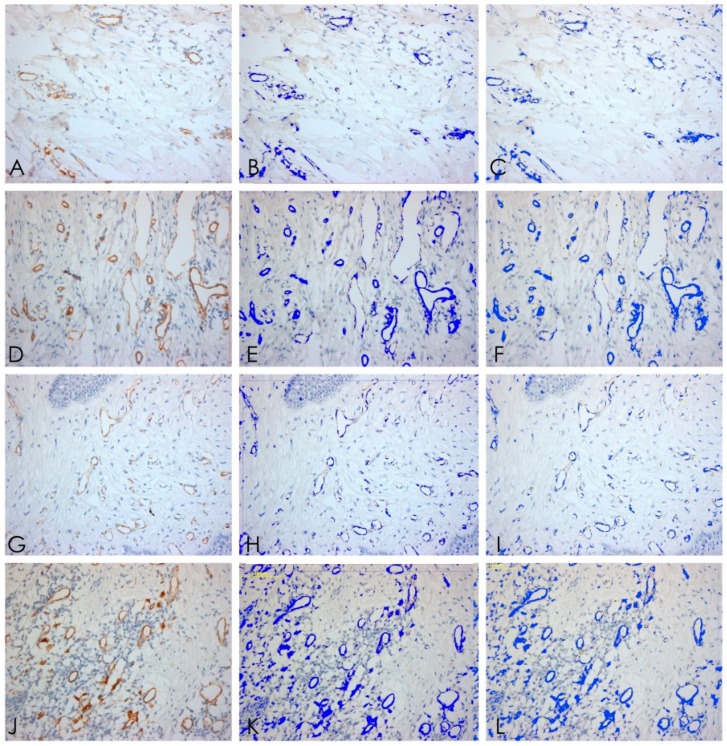
CD31. (**A**–**C**) Vessels expression T1, 20× ABC; and software analysis; (**D**–**F**) vessels expression T2, 20× ABC, and software analysis; (**G**–**I**) vessels expression T3, 20× ABC, and software analysis; (**J**–**L**) vessels expression T4, 20× ABC, and software analysis.

The samples examined appeared clinically healthy with no plaque accumulation, suppuration, or bleeding on probing. The results from the immunohistochemical investigation showed the presence of adhesion molecules in contact with the HA, in all samples, although in a different way. It is important to underline that all the samples, even if clinically healthy, were positive for the different clusters related to the inflammatory infiltrate. The immunohistochemical results of α6 integrins are depicted in Figure 2; in every image, the right part represents the soft tissue side close to HAs. By investigating the different surfaces of the HAs, it was shown that the infiltrate was minimal for T1 samples (DMLS surface) compared with T4 (entirely machined surface) samples, with a statistically significant difference (*p* < 0.001) (Figure 3). The specific inflammatory infiltrate was composed of T lymphocytes (CD3), B lymphocytes (CD20), and macrophages (CD68). It predominantly consisted of lymphocytes B. Regarding the extension, differences between groups were found. These results of inflammatory infiltrate and microvessel density (MDV) are shown in Figure 4, Figure 5, Figure 6 and Figure 7. With regard to the adhesion molecules, the samples showed different intensity of expression, statistically significant (*p* < 0.001) between T1 and the other groups. The specific results for the different test groups are reported below.

### 3.1. Healing Abutments with Full Direct Metal Laser Sintering Surface (T1)

The samples of the T1 group (totally DMLS) presented the higher expression of integrins. At the same time, they presented lower values of inflammatory infiltrate and vessel density. Expression of integrins was 25.56 ± 3.11%. T lymphocytes (CD3) constituted 0.13 ± 0.01% of the inflammatory infiltrate, the percentage of B lymphocytes was 0.16 ± 0.01%, and the macrophages (CD68) constituted 0.43 ± 0 03%. It was found also that the average number of vessels was 13.49 ± 0.63 per mm² for each acquisition field; considering that each sample had five acquisition fields, the average density was 67.45 ± 0.63 per mm².

### 3.2. Healing Abutments with Smooth Surface in the Upper Half and Direct Metal Laser Sintering Surface in the Lower Half (T2)

In these samples, integrins expressed themselves with a percentage of 8.88 ± 1.51%. The inflammatory infiltrate was composed by T lymphocytes (CD3) 1.45 ± 0.14%; B lymphocytes (CD20) 3.01 ± 0.40%; and macrophages constituted 2.65 ± 0.39%. The number of vessels was equal to 25.4 ± 0.73 per mm², equal to 127 ± 0.73 per mm² considering the five acquisition fields.

### 3.3. Healing Abutments with Direct Metal Laser Sintering Surface in the Upper Half and Smooth Surface in the Lower Half (T3) 

In these samples, the integrins expression was 16.64 ± 6.73%. Within these samples, there was an inflammatory infiltrate consisting of T lymphocytes (CD3) 0.93 ± 0.07%, B lymphocytes (CD20) 0.75 ± 0.05%, while the macrophages constituted 1, 04 ± 0.07% and the density of the vessels was 17.04 ± 0.58 per mm² equal to 85.2 ± 0.58 per mm for the five acquisition fields.

### 3.4. Healing Abutments with Machined Surface (T4)

These samples formed by totally machined healing screws showed higher values of inflammatory infiltrate and vessels and lower values of integrins. In particular, the latter presented values of 7.04 ± 4.26%. While values of 3.90 ± 0.31% were shown for T lymphocytes (CD3); the B lymphocytes (CD20) were equal to 7.86 ± 0.54%; macrophages constituted 4.5 ± 0.27%. Examining the density of the vessels, there was a value of 30.32 ± 0.79 per mm² equal to 151.6 ± 0.79 per mm² for the five acquisition fields.

**Figure 8:**
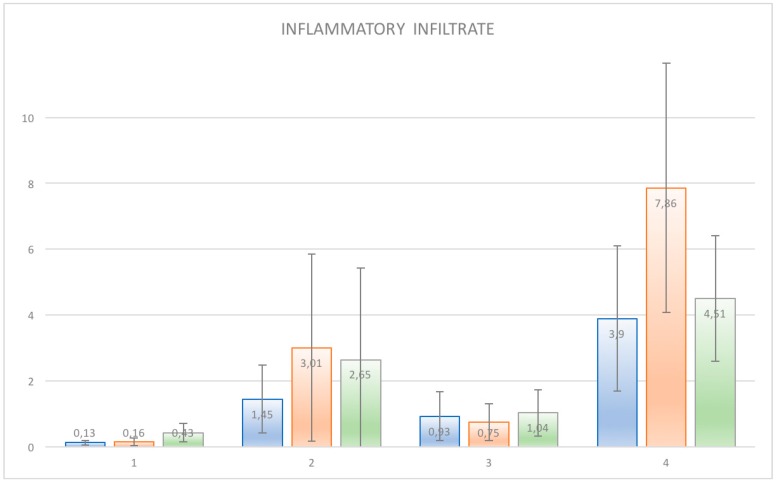
Graphical representation of different markers expression. Markers were evaluated individually. Statistical significant differences between groups were find. Inflammatory infiltrate evaluated by CD3 (in blue), CD20 (orange), and CD68 (green).

**Figure 9:**
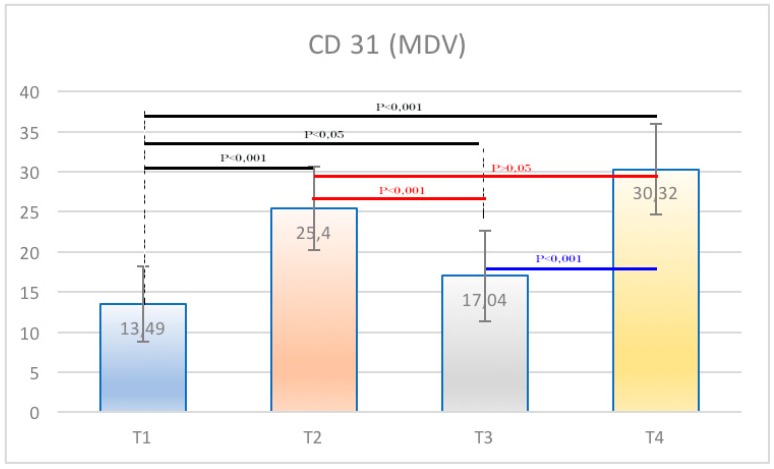
Graphical representation of different CD31 expression. Statistical significant differences between groups were find. MDV—microvessel density.

Finally, the percentage of expression of B lymphocytes from the immunohistochemical analysis was the most significant value for the inflammatory infiltrate given the highest concentration of these cells compared with the others in T4 and the very low percentage in T1 with highly significant results (*p* < 0.001). With regard to integrins, statistically significant results emerged in the comparison between the T1 groups and all the other groups. The data were statistically analyzed and reported as mean values with standard deviation and standard error (Table 1 and Table 2). Furthermore, for each cell population and vessel density, the various samples were compared by Dunn’s multiple comparison test. The results showed statistically significant differences between the groups (*p* < 0.001) (Table 3, Table 4, Table 5, Table 6, Table 7, Table 8, Table 9 and Table 10) (Figure 8 and Figure 9).

## 4. Discussion

Since the advent of implant rehabilitation, a considerable interest has been developed in evaluating and analyzing how hard tissues (bone in particular) interact with these medical devices [36,37]. In a few years, the literature has provided a huge number of results on the ability of bone tissue cells to bind titanium with huge variables depending on the implant surface treatment [25,38,39]. On the other hand, a minor interest has been directed to the effects on the transmucosal portion provided by surface treatments and different materials [1,18,20,40].

Until now, in implantology, the scientific literature has not clarified whether it is preferable to have a transgingival implant portion with controlled roughness or a smooth (machined) one [40].

Some authors believe that a transgingival portion with a machined surface may reduce the adhesion of bacteria and the consequent risk of bacterial colonization and inflammation of soft and hard tissues around the implant [41,42]. According to this point of view, a machined implant collar and a smooth transgingival portion should be able to reduce the incidence of peri-implantitis; but peri-implantitis remains today a mysterious pathology with multifactorial characteristics [43].

Moreover, these observations are mostly based on in vitro studies of bacterial adhesion to different surfaces, therefore, the evidence that emerges is rather low, and cannot be directly translated in vivo [43].

On the other hand, some authors believe that a rough surface can promote epithelial and connective adhesion, and thus represent an ideal solution, tending to favor the formation of a mucosal barrier, useful to counteract bacterial penetration [1,18,19,20,21]. This soft tissue barrier may reduce the risk of peri-implantitis.

In a previous immunoistochemical study [44], the authors aimed to characterize the cellular and molecular patterns for bone loss of soft tissues surrounding implants restored with different implant platform configurations. In total, 32 fixtures were restored with abutments with different mismatches: 0 mm, 0.25 mm, 0.5 mm, and 0.85 mm [44]. Four years later, all sites were clinically healthy, and soft tissue samples were harvested for immunohistochemical analysis [44]. In addition, no significant difference was found between groups in terms of cells infiltrate, then the authors concluded that the configuration of the implant abutment interface does not seem to affect the inflammatory cellular and molecular pattern responsible for bone loss [44].

In the present study, the presence of a sufficiently large proportion of keratinized gingiva guaranteed a good healing of the tissues in the post-surgical phase (inclusion criteria). Furthermore, in all specimens, inflammatory infiltrate levels were acceptable, though with significant differences. It was also important to underline how the re-use of HAs among different patients, mainly for economic reasons, may have effects on the early inflammation of the peri-implant tissues linked to the residues present after the common sterilization cycles. In two different studies [45,46], authors studied the effect of re-use of HAs in clinical practices. Wadhwani et al. [45] showed that the healing screws treated according to the standard cleaning and sterilization protocols for subsequent uses showed residues protein contamination at one or more sites in 90% of cases. Also, Stacchi et al. [46] demonstrated a better cell binding in HAs treated with specific cleaning methods compared with the control re-used screws and treated with standard methods. Our results obtained with new HA demonstrated an excellent level of integration and a low level of peri-implant inflammation. We could speculate that the use of a new healing abutment every time in order to avoid contamination on surface.

However, the main interest was thus to provide a surface able to positively influence cell binding, and the immunohistochemical analysis demonstrated the presence of adhesion molecules like integrins between the HAs and the peri-implant tissues. These molecules were distributed differently depending on the type of surface treatment. Specifically, in HAs with DMLS surface, the presence of integrins had been found to be significantly higher (*p* < 0.001) than that in HAs with machined surface. These findings demonstrate that a predetermined and controlled roughness performed by DMLS methods can induce the formation of more hemidesmosomes, thus ensuring the formation of a stable bond between the soft tissues and the HA. The more the constituent proteins of these structures are expressed, the greater the adhesion that the epithelium manages to contract with the device and the more effective the sealing action carried out by the soft tissues will be. The “barrier” role of the peri-implant epithelium was thus guaranteed to be the best and represented an additional control to the passage of bacteria from the oral cavity to the underlying hard tissues. In the current scientific literature, other studies showed that the integration of epithelial cells occurred indifferently on treated or machined surfaces [47,48].

From our present study, therefore, it was evident that porous surfaces, with a proper roughness (of appropriate size in terms of depth and ridges), can improve and promote soft tissue adhesion rather than hinder it. Considering such scientific evidence, it should be reasonable to assume that producing a porous surface with methods such as DMLS may allow one to obtain a predefined surface topography in each single point, with favorable histological outcomes, as previously evidenced in another human soft tissue study [34]. Therefore, it is not the roughness itself that impedes or promotes the epithelial adhesion, but it is the type of roughness that influences the behavior of cells [34]. In this regard, several studies in literature showed, on the contrary, that the machined surface can positively influence epithelial growth [49,50]. Authors [49] showed how the epithelial growth was reduced in the rough tested surfaces. In another study [50], it was shown that fibroblasts preferred the rough surfaces, while the epithelial cells did not. In our study, the type of treatment, DMLS, and the regularity of the roughness obtained can justify this seemingly contrasting result.

In the present study, the presence of an inflammatory infiltrate was found in all the samples under examination. Specifically, the inflammatory infiltrate consisted of lymphocytes, plasma cells, and macrophages. A higher value of T lymphocytes (CD3+) and B lymphocytes (CD20+) was observed in the specimens of the T4 group. A higher value of MDV (microvessel density) was also found in T4 compared with T1. This also agrees with the results reported by Artese et al. [18], which showed an increased expression of VEGF (vascular endothelial cell growth factor) linked to a major inflammation. Angiogenesis is implicated in both the repair and inflammation processes [24]. The literature has shown that the vascular network increased during increased inflammation and edema [24]. In the present study, the increase in vessel density, found on machined surfaces, coincided with an increase of inflammatory cells.

## 5. Conclusions

The present study aimed to evaluate cell adhesion and the inflammatory infiltrate on HAs with different surface characteristics (fully porous DMLS, half DMLS and half machined, fully machined). The immunohistochemical analysis demonstrated the presence of adhesion molecules (integrins) between the HAs and the peri-implant tissues. These molecules were distributed differently depending on the type of surface treatment, and in the HAs with DMLS surface, the presence of integrins had been found to be significantly greater than those found on the machined surface. Finally, in this study, all samples were positive for the different clusters related to the inflammatory infiltrate (T lymphocytes, CD3; B lymphocytes, CD20; and macrophages, CD68), but a statistically significant lower infiltrate was found in HAs with DMLS surface, when compared with Has with machined surface. In conclusion, the HA surface seems to influence the degree of cell adhesion and the inflammatory infiltrate of the surrounding soft tissues. The results of the present study are the first to demonstrate the excellent performance of DMLS surface applied to HA. For this reason, further studies should be performed in order to extend the sample size. Also, a longer follow-up could clarify the behavior during the function of restoration. Finally, the same investigation could be performed using this surface treatment in the transmucosal portion of the prosthetic abutment to evaluate its effectiveness during the masticatory load.

## Figures and Tables

**Table 1:** Mean and SD of integrins expression in different groups.

	T1	T2	T3	T4
mean	26.13	13.03	16.64	7.04
SD	3.11	4.47	6.73	4.26

**Table 2:** Integrin expression Dunn’s multiple comparison between groups.

Comparison	*p* Value
T1 vs. T2	<0.001
T1 vs. T3	<0.001
T1 vs. T4	<0.001
T2 vs. T3	<0.05
T2 vs. T4	<0.01
T3 vs. T4	<0.001

**Table 3:** Mean and SD of T Lymphocytes (CD3).

	T1	T2	T3	T4
mean	0.13	1.45	0.93	3.90
SD	0.07	1.03	0.74	2.20

**Table 4:** CD3 Dunn’s multiple comparison between groups.

Comparison	*p* Value
T1 vs. T2	<0.001
T1 vs. T3	<0.001
T1 vs. T4	<0.001
T2 vs. T3	>0.05
T2 vs. T4	<0.001
T3 vs. T4	<0.001

**Table 5:** Mean and SD of B Lymphocytes (CD20).

	T1	T2	T3	T4
mean	0.16	3.01	0.75	7.86
SD	0.11	2.84	0.56	3.78

**Table 6:** CD20 Dunn’s multiple comparison between groups.

Comparison	*p* Value
T1 vs. T2	<0.001
T1 vs. T3	<0.001
T1 vs. T4	<0.001
T2 vs. T3	<0.001
T2 vs. T4	<0.01
T3 vs. T4	<0.001

**Table 7:** Mean and SD of Macrophages (CD68).

	T1	T2	T3	T4
mean	0.43	2.65	1.04	4.51
SD	0.28	2.79	0.70	1.91

**Table 8:** CD68 Dunn’s multiple comparison between groups.

Comparison	*p* Value
T1 vs. T2	<0.001
T1 vs. T3	<0.001
T1 vs. T4	<0.001
T2 vs. T3	<0.001
T2 vs. T4	<0.001
T3 vs. T4	<0.001

**Table 9:** Mean and SD of microvessel density (MDV) (CD31).

	T1	T2	T3	T4
mean	13.49	25.40	17.04	30.32
SD	4.69	5.22	5.66	5.62

**Table 10:** MDV (CD31) Dunn’s multiple comparison between groups.

Comparison	*p* Value
T1 vs. T2	<0.001
T1 vs. T3	<0.05
T1 vs. T4	<0.001
T2 vs. T3	<0.001
T2 vs. T4	> 0.05
T3 vs. T4	<0.001
